# Supplementary material for: A high-resolution mRNA expression time course of embryonic development in zebrafish
Source: eLife. 2017 Nov 16;6:e30860. doi: 10.7554/eLife.30860 (PMC5690287; doi:10.7554/eLife.30860)
Supplement: Supplementary file 6. [file elife-30860-supp6.zip › biolayout-clusters-files/Cluster106-genes.html]

Cluster106


# Cluster106: Genes

| Ensembl ID | Gene Name | Chr | Start | End | Biotype |
| --- | --- | --- | --- | --- | --- |
| ENSDARG00000101139 | agpat2 | 21 | 7627452 | 7661673 | protein\_coding |
| ENSDARG00000061196 | emilin2a | 2 | 31001409 | 31037320 | protein\_coding |
| ENSDARG00000014239 | esyt1b | 6 | 52262927 | 52339927 | protein\_coding |
| ENSDARG00000075564 | fam13a | 1 | 50050973 | 50147451 | protein\_coding |
| ENSDARG00000059246 | hnrnpd | 10 | 5000319 | 5016693 | protein\_coding |
| ENSDARG00000031336 | hsd20b2 | 5 | 37600654 | 37614654 | protein\_coding |
| ENSDARG00000076520 | mafb | 25 | 36103766 | 36106032 | protein\_coding |
| ENSDARG00000060235 | mst1ra | 6 | 42478194 | 42508668 | protein\_coding |
| ENSDARG00000043243 | prkchb | 20 | 20463810 | 20510454 | protein\_coding |
| ENSDARG00000022904 | skilb | 24 | 26225653 | 26241586 | protein\_coding |
| ENSDARG00000005463 | slc30a1a | 20 | 13998143 | 14017810 | protein\_coding |
| ENSDARG00000037012 | slc3a2b | 21 | 26684674 | 26689182 | protein\_coding |
